# Evaluating implementation of “management of Possible Serious Bacterial Infection (PSBI) when referral is not feasible” in primary health care facilities in Sindh province, Pakistan

**DOI:** 10.1371/journal.pone.0240688

**Published:** 2020-10-14

**Authors:** Maria Bhura, Shabina Ariff, Shamim Ahmad Qazi, Zaitoon Qazi, Imran Ahmed, Yasir bin Nisar, Zamir Suhag, Abdul Wahab Soomro, Sajid Bashir Soofi

**Affiliations:** 1 Center of Excellance in Women & Child Health, Aga Khan University, Karachi, Pakistan; 2 Department of Paediatrics and Child Health, Aga Khan University, Karachi, Pakistan; 3 Retired from Department of Maternal, Newborn, Child, and Adolescent Health, WHO, Working as a WHO Consultant, Geneva, Switzerland; 4 Independent Consultant, Geneva, Switzerland; 5 Department of Maternal, Newborn, Child, Adolescent Health and Aging, World Health Organization, Geneva, Switzerland; 6 People’s Primary Healthcare Initiative, Sindh, Pakistan; Medical Research Council, SOUTH AFRICA

## Abstract

**Background:**

The World Health Organization (WHO) launched a guideline in 2015 for managing Possible Serious Bacterial Infection (PSBI) when referral is not feasible in young infants aged 0–59 days. This guideline was implemented across 303 Basic Health Unit (BHU) Plus primary health care (PHC) facilities in peri-urban and rural settings of Sindh, Pakistan. We evaluated the implementation of PSBI guideline, and the quality of care provided to sick young infants at these facilities.

**Methods:**

Thirty (10%) out of 303 BHU Plus facilities were randomly selected for evaluation. A survey team visited each facility for one day, assessed the health system support, observed the management of sick young infants by health care providers (HCP), validated their management, interviewed HCPs and caretakers of sick infants. HCPs who were unable to see a young infant on the day of survey were evaluated using pre-prepared case scenarios.

**Results:**

Thirty (100%) BHU Plus facilities had oral amoxicillin, injectable gentamicin, thermometers, baby weighing scales and respiratory timers available; 29 (97%) had disposable syringes and needles; 28 (93%) had integrated management of childhood illness (IMCI)/PSBI chart booklets and job aids and 18 (60%) had a functional ambulance. Each facility had at least one HCP trained in PSBI, and 21 (70%) facilities had been visited by a supervisor in the preceding six months. Of 42 HCPs, 19 (45.3%) were trained within the preceding 12 months. During the survey, 26 sick young infants were identified in 18 facilities. HCPs asked about history of breastfeeding in 23 (89%) infants, history of vomiting in 17 (65%), and history of convulsions in 14 (54%); weighed 25 (97%) infants; measured respiratory rate in all (100%) and temperature in 24 (92%); assessed 20 (77%) for movement and 14 (54%) for chest indrawing. HCPs identified two infants with fast breathing pneumonia and managed them correctly per IMCI/PSBI protocol. HCPs identified six (23%) infants with clinical severe infection (CSI), two of them were referred to a higher-level facility, only one accepted the referral advice. Only one CSI patient was managed correctly per IMCI/PSBI protocol at the outpatient level. HCPs described the PSBI danger signs to eight (31%) caretakers. Caretakers of five infants with CSI and two with pneumonia were not counselled for PSBI danger signs. Five of the six CSI cases categorized by HCPs were validated as CSI on re-examination, whereas one had pneumonia. Similarly, one of the two pneumonia patients categorized by HCPs had CSI and one identified as local bacterial infection was classified as CSI upon re-examination.

**Conclusion:**

Health system support was adequate but clinical management and counselling by HCPs was sub-optimal particularly with CSI cases who are at higher risk of adverse outcomes. Scaling up PSBI management is potentially feasible in PHC facilities in Pakistan, provided that HCPs are trained well and mentored, receive refresher training to appropriately manage sick young infants, and have adequate supplies and counselling skills.

## Introduction

Of 5.3 million estimated child deaths in 2018, 2.5 million occurred in the neonatal period [1]. Infections, intrapartum complications, and preterm birth account for most of these deaths. In 2016, neonatal infections including pneumonia, sepsis and meningitis were responsible for 21% of neonatal deaths annually and were responsible for nearly 550,000 neonatal deaths, nearly all of them occurring in developing countries [2].

The standard practice to manage young infants with any sign of Possible Serious Bacterial Infection (PSBI) is in a hospital setting with parenteral antibiotics, which may not be feasible in low resource settings, especially in the low- and middle-income countries (LMIC). Simplified regimens comprising of injectable plus oral antibiotics delivered outside the hospital setting when referral was not feasible, were shown to be effective in India [3], Bangladesh [4] and in Pakistan [5]. Later, four large community-based trials were designed in settings where referral was not feasible in five African and Asian countries (Bangladesh, Democratic Republic of Congo, Kenya, Nigeria and Pakistan) [6–9]. Their results showed that barring critically ill young infants, simplified antibiotic regimens could safely and effectively treat young infants with signs of PSBI when referral was not feasible. The above evidence contributed to the development of the World Health Organization (WHO) guideline for management of young infants with signs of PSBI when referral is not feasible [10].

Pakistan’s health system at the district level has Basic Health Units (BHU) as the first level health facility in the primary health care (PHC) structure, catering for a population of between 5000 to 10,000 people. Five to six BHUs are linked with a Rural Health Centre (RHC), which caters for around 30,000 to 50,000 population. Above these are the referral units at Sub-district and district hospital level, followed by tertiary care hospitals.

The People’s Primary Healthcare Initiative (PPHI) in the province of Sindh, is a public-private partnership programme of the Government of Sindh. PPHI Sindh manages 1176 PHC facilities in all districts across Sindh, which cover over half the population (25.9 million) of the Sindh province (47.9 million)and 58% of all public PHC facilities [11]. The organization’s main focus is improving health care in the area of maternal, newborn and child health which includes antenatal care, labour and delivery, postnatal care, family planning, immunization, nutrition, basic emergency obstetric and newborn care (BEmONC), diagnostic laboratories, ambulance service etc.

PPHI Sindh and Aga Khan University (AKU), Pakistan implemented WHO’s PSBI guideline when referral was not feasible in 10 Basic Health Units (BHUs) in Thatta and Sujawal districts of Sindh province, Pakistan from May 2016 to September 2017. In the implementation research area, around 12% of the young infants brought to a health facility had signs of PSBI and over 80% of the families refused referral to the hospital in young infants who had signs of PSBI that required referral (unpublished data). Similar findings were seen in Bangladesh, where around 11% of infants visiting health facilities were identified with PSBI [12]. Majority of sick young infants with signs of PSBI were successfully treated in these BHUs with simplified antibiotic regimens (oral amoxicillin for 7 days plus injectable gentamicin for 2 days) when families refused referral. In addition, all young infants 7–59 days of age with fast breathing pneumonia only were treated effectively at BHUs with oral amoxicillin for 7 days (unpublished data).

PPHI scaled up the management of PSBI in 303 BHU Plus facilities (compared to a BHU, a BHU Plus facility delivers PHC services round the clock (24/7) in the Sindh province, using the updated chart booklet and related training materials [13]. The scale-up included identification and training of healthcare providers (HCPs) who managed sick young infants at health facilities. Their theoretical and hands on training included PSBI management i.e., assessment of sick young infants, classification of illness, referral if needed, pre-referral treatment, treatment when referral was not feasible, injection safety and counselling of parents. A supply chain was also set in place by PPHI to provide and distribute essential commodities, job aids and PSBI registries at these facilities.

AKU in collaboration with PPHI conducted study to evaluate the quality of PSBI management guideline implementation in the PPHI health facilities.

## Methods

### Objectives

The objectives of the health facility survey were to i) evaluate the support provided by the health system, such as health facility staffing, staff training, availability of drugs and equipment and supervision; ii) determine how sick infants were assessed at outpatient health facilities according to PSBI guidelines by healthcare providers; iii) assess the quality of counselling provided at outpatient health facilities and its understanding by caretakers for home treatment of their sick infant.

The survey was conducted in 30 (10%) randomly selected facilities from 303 BHU Plus facilities, which included six in Larkana division, nine in Sukkur division, seven in Mirpurkhas division and eight in Hyderabad division. Twenty-four (80%) facilities were in rural areas and six (20%) were in peri-urban localities. Non-stratified randomization was conducted through a randomization code generated using STATA v.15. The survey instruments were developed using health facility evaluation methodology prepared by the WHO [14] and included, i) observation checklist; ii) exit interview–caretaker of the child; iii) re-examination of the sick young infant; iv) equipment and supply checklist; and v) case scenarios (when there was no sick young infant).

These instruments were pre-tested, and surveyors were trained on their use. The surveyors were non-medical personnel, experienced in health facility assessments, and had a Master degree. They were locals who spoke Sindhi, Urdu and English and did not require an interpreter as they were well versed in the local languages. Each team had two surveyors, who compared their findings at the end of each working day and no discrepancies were found among their findings. All surveyors were trained in the management of PSBI including identifying various PSBI signs in sick young infants, and their management through theoretical orientation and practical skill sessions. Two survey teams with two surveyors in each team were allocated to two provincial divisions per team. The survey was conducted from 26^th^ June to 13^th^ July 2019 after obtaining informed written consent from the infant’s caretaker and the health care provider (HCP).

Sick young infants aged 0–59 days who presented at any selected health facility during the survey were enrolled and the survey teams observed the consultation between the caretaker of sick infant and the HCP, which included assessment of PSBI signs, classification of illness, treatment prescribed, referral advice and counselling regarding danger signs and infant care at home. The team also conducted an exit interview with the caretakers of the sick infants to assess their understanding of danger signs and their perception of services provided by the health facility. Subsequently, the surveyors re-examined and classified the sick young infant using the PSBI clinical guideline (Panel 1). The team assessed the health facility for commodities (medicines and equipment) required to treat PSBI cases. They interviewed the HCP about their training/refresher training and supervision status. When no young infant was available on the day of the survey, five PSBI case scenarios (15 questions per scenario) were used to evaluate the HCPs for their ability to diagnose, classify, treat, refer a PSBI case and counsel parents. Questions were divided into four categories i.e., diagnosis, management, counselling, and referral of PSBI. Each correct answer was scored as 1 and incorrect answer as 0.

**Panel 1:** PSBI classification list.

PSBI Classification	Signs and Symptoms	Management/Treatment
Critical Illness	Having at least one sign of the following: convulsions, unable to feed at all, does not move even on stimulation, unable to cry at all, cyanosis, bulging fontanelle	Referral for hospitalization after pre-referral treatment (one dose of benzyl penicillin 50 000 IU/kg and gentamicin 5–7.5mg/kg)
Clinical Severe Infection	Having at least one sign of severe infection (i.e. movement only when stimulated, stopped feeding well on observation, temperature ≥ 38°C or ≤ 35.5°C, 0–6 days old infant with respiratory rate of ≥ 60 breaths per minute or severe chest in-drawing).	Referral for hospitalization after pre-referral treatment. If refused, give intramuscular gentamicin 5–7.5 mg/kg once daily for two days and twice daily oral amoxicillin, 50 mg/kg per dose for seven days
Pneumonia	7–59 days old infant with respiratory rate of ≥ 60 breaths per minute	Oral amoxicillin, 50 mg/kg per dose twice daily for seven days

PSBI classifications with their associated signs/symptoms and the required management/treatment in infants aged 0–59 days. Adapted from ([10])

Data was collected on paper-based instruments and entered manually into Microsoft Excel. The data was then rechecked with the forms by the survey coordinator. The data was analyzed in STATA v.15 using the priority indicators list developed in consultation with WHO. Exploratory analyses were conducted for the priority indicators and were presented as frequencies and percentages.

### Ethics approval and consent

"This study was an extension of a previous study that received approval from the Ethical Review Committee at the Aga Khan University, ERC Number: 3936-Ped-ERC-15-PI Sajid Soofi. Participants and healthcare providers provided informed, signed consent before participating in the study. Those who were unable to sign provided a thumb impression to express consent".

## Results

### Health facilities

All 30 BHU Plus health facilities had oral amoxicillin, injectable gentamicin, thermometers, baby weighing scales and respiratory timers available (Table 1). Except one, all (97%) facilities had disposable syringes and needles, 28 (93%) had antiseptic swabs. The integrated management of childhood illness (IMCI)/PSBI chart booklets and job aids were present in 28 (93%) facilities. Five (17%) facilities had an antibiotic stockout in the past one month whereas 6 (20%) had a stockout within the past three months. An ambulance was available in 18 (60%) facilities to transport very sick infants to higher level facilities (Table 1). Twenty-one (70%) facilities had received at least one supervisory visit in the preceding six months that included observation of case management (Table 1). Twenty-four (80%) health facilities routinely, received referred infants from affiliated lady health workers (LHWs), whereas 25 (83%) health facilities routinely referred sick infants to higher-level health facilities. In the one month prior to the survey, 20 (66.7%) health facilities had referred sick young infants to higher level facilities. Moreover, 25 (83.3%) facilities had registries with PSBI case classifications of infants visiting.

**Table 1:** Indicators for health system support, availability of commodities and referral mechanism at health facilities (n = 30).

Health facility indicators	n (%)
Health facilities that received at least one supervisory visit including observation of case management during the previous six months	21 (70%)
Health facilities with at least one health worker trained in management of young infants with PSBI	30 (100%)
Health facilities with a stockout of medicines in the past one month	5 (16.7%)
Health facilities with a stockout of medicines in the past three months	6 (20%)
Health facilities where staff held meetings to discuss PSBI cases	28 (93.3%)
Health facilities where lady health workers referred infants	24 (80%)
Health facilities that referred PSBI cases to other higher-level facilities	25 (83.3%)
Health facilities that referred a PSBI case in the month prior to the survey	20 (66.7%)
Health facilities with a PSBI case classification registry	25 (83.3%)
**Availability of commodities at the health facility**	
Oral amoxicillin	30 (100%)
Injectable gentamicin	30 (100%)
Thermometers	30 (100%)
Weighing scale	30 (100%)
Respiratory timer	30 (100%)
Disposable syringes and needles	29 (96.7%)
Antiseptic swabs	28 (93.3%)
IMCI chart booklet/PSBI job aid	28 (93.3%)
Ambulance service	18 (60%)

We identified 42 HCPs across the 30 health facilities who were trained in PSBI management and were involved in newborn and child health services, out of which 40 (95.2%) were qualified physicians (medical officers) and two were lady health visitors (skilled birth attendants) (Table 2). Nineteen (45.2%) HCPs received their PSBI management training within the past year (Table 2). The number of sick young infants (0–59 days of age) and those with PSBI signs visiting the health facilities monthly varied (Table 2).

**Table 2:** Descriptive characteristics of HCPs (n = 42) and PSBI cases at health facilities (n = 30).

Characteristics	n (%)
**Classification of healthcare providers**	
Medical physicians	40 (95.2%)
Other HCPs	2 (4.8%)
**Time since PSBI training received**	
<6 months	2 (4.8%)
6-<12 months	17 (40.5%)
12-<24 months	18 (42.9%)
24+ months	5 (11.9%)
**Number of sick young infants (0–59 days) visiting the health facility (monthly)**	
0–10	5 (16.7%)
11–20	6 (20%)
21–50	12 (40%)
51–100	4 (13.3%)
100+	3 (10%)
**Number of PSBI cases (0–59 days of age) identified at the health facility the month prior to the survey**	
None	4 (13.3%)
1–10	11 (36.7%)
11–20	5 (16.7%)
21–50	9 (30%)
>50	1 (3.3%)
**Number of sick young infants (0–59 days of age) brought by the families to a health facility during the survey**	26
Number of health facilities where sick young infants were brought on the day of the survey	18 (60%)

### Assessment and management of sick, young infant by healthcare provider

**Fig 1:**
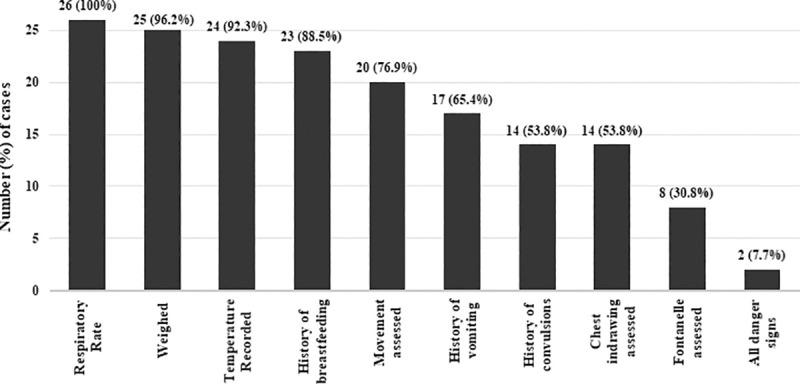
Assessment of sick young infants by health care providers for PSBI danger signs (n = 26).

Twenty-six young infants aged 0–59 days were identified in 18 facilities where six (23%) were between 0–6 days of age and 20 (77%) were between 7–59 days of age. In the 18 facilities mentioned above, 21 (81%) HCPs used the IMCI chart booklet/PSBI job aid while assessing young infants. HCPs asked 23 (89%) caretakers about history of breastfeeding and if their infant was feeding well or not, 17 (65%) about history/occurrence of vomiting and 14 (54%) about history of convulsions. HCPs weighed 25 (97%) infants, measured respiratory rate in all 26 infants, measured temperature in 24 (92%), assessed 20 (77%) for sign of no or limited movement, 14 (54%) for severe chest indrawing, and checked 8 (31%) for bulging of fontanelle. Only two (8%) infants were assessed for all PSBI danger signs by the HCPs who examined them (Fig 1).

**Fig 2:**
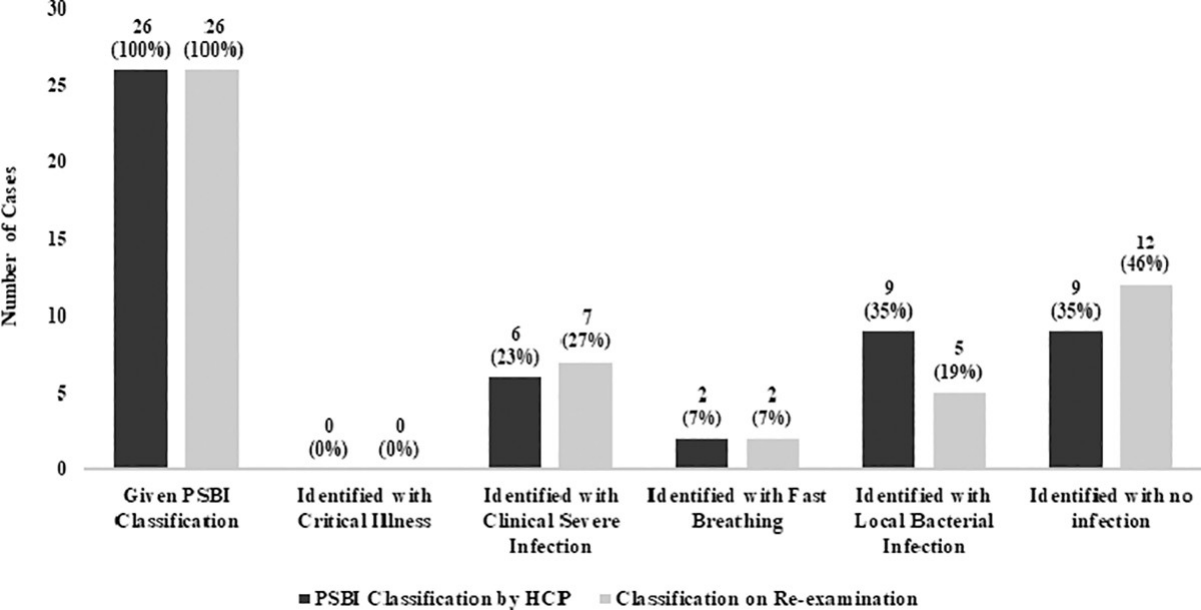
Identification of PSBI in infants aged 0–59 days by the HCP and on re-examination by surveyor (n = 26).

Among 26 sick young infants who visited health facilities at the time of survey, HCPs did not classify any child with critical illness, classified six (23%) with clinical severe infection (CSI), two with fast breathing pneumonia, nine with local bacterial infection and nine with no signs of infection (Fig 2). Both infants identified with pneumonia were prescribed oral amoxicillin in correct dose and frequency to be taken at home according to the IMCI/PSBI protocol. Of six CSI cases, two were referred to a higher-level facility but only one family accepted the referral advice. The infant who accepted referral was not given pre-referral antibiotics. Moreover, only one out of six infants with CSI was advised the correct antibiotics in correct dose and none were advised in correct frequency by the HCPs as per IMCI/PSBI protocol. On validation, surveyors re-examined 26 infants and classified seven (27%) infants with CSI, two with pneumonia, five as local bacterial infection and 12 (46%) infants with no signs of infection (Fig 2). However, on re-examination, of six young infants categorized as CSI cases by HCPs, five were validated as CSI and one infant only had pneumonia. Similarly, on re-examination, one of the two cases of pneumonia categorized by HCPs had CSI. In addition, of nine cases categorized as local infection by HCPs five were validated as local infection on re-examination, whereas one as CSI and three as no infection.

### Counselling of caretakers

HCPs counselled 24 (92%) caretakers to continue exclusive breastfeeding, advised 21 (81%) to bring the infant back if their condition worsened, and 14 (54%) were advised to keep the infant warm. HCPs described the PSBI danger signs to eight (31%) caretakers to watch out for, out of which two had CSI, two had local infection and four had no infection. Caretakers of four infants with CSI and two with pneumonia were not counselled for danger signs by HCPs.

### Case scenario assessment

**Fig 3:**
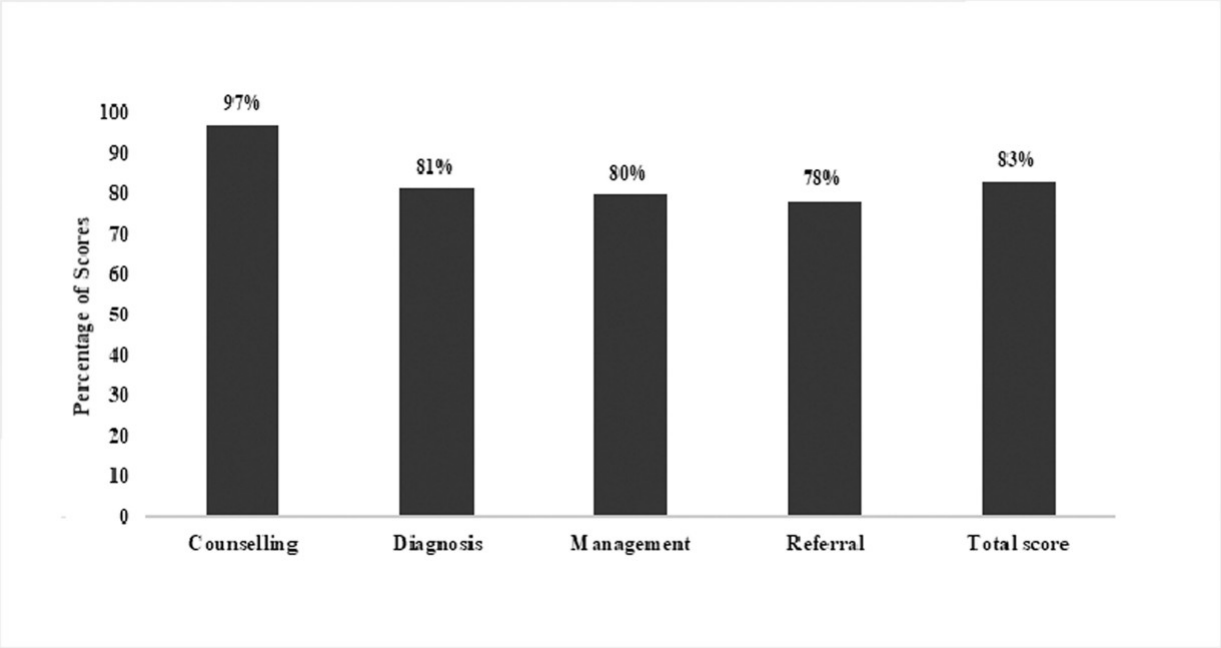
Average scores in percent for health care providers (n = 40) assessment through 78 case scenarios across PSBI management categories.

When no young infant was present at the facility on the survey visit day for the HCP to assess, they were evaluated on case scenarios. We recorded results from 78 case scenarios (Fig 3). HCPs scored 97% on an average on questions regarding counselling, 81% on PSBI diagnosis, 80% on disease management and 78% on referral. The average total score of HCPs at PSBI health facilities was 83%. Healthcare providers who observed none of only one infant during the survey visit responded to case scenarios to assess their knowledge of PSBI across four categories: diagnosis, treatment, referral and management. Two HCPs did not answer case scenarios since they observed two infants each.

### Exit interview of caretakers

**Fig 4:**
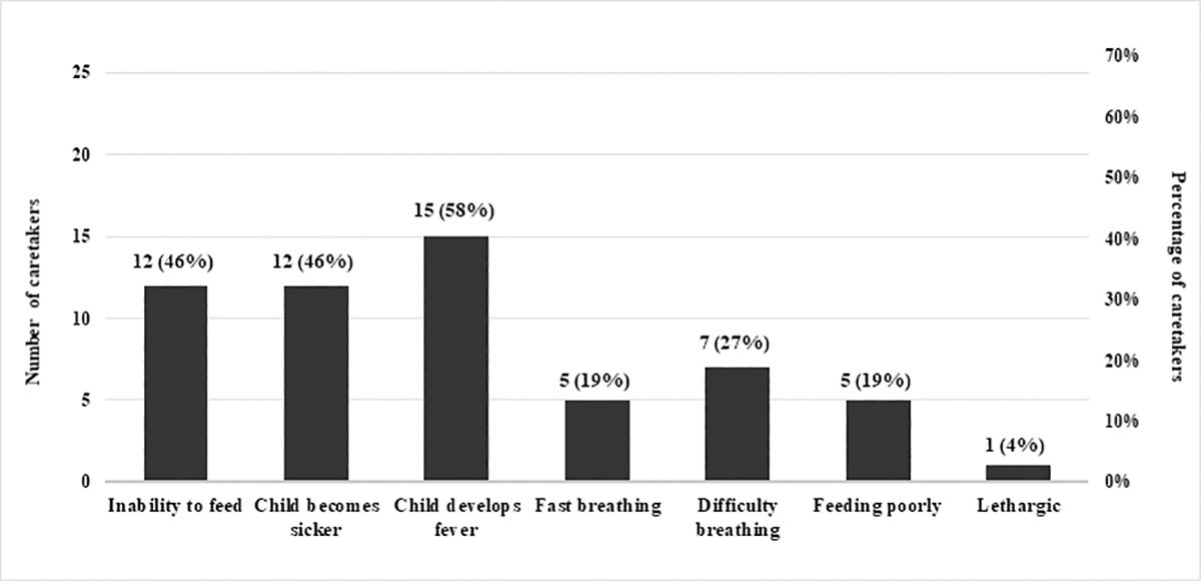
Caretakers’ understanding of danger signs (n = 26).

Twenty-six caretakers were interviewed, including 19 mothers, one father and six other relatives. During the exit interview, surveyors asked caretakers to describe symptoms in an infant that would prompt them to bring their infant to a health facility (Fig 4). Despite visiting HCPs, caretakers’ knowledge of danger signs was poor. The responses varied, ranging from 15 (58%) reporting fever as a danger sign, 12 (46%) reporting inability to feed and “becomes sicker” to one (4%) reported lethargy as a danger sign. Less than a quarter of participants reported fast breathing (19%) and feeding poorly (19%) as danger signs. Caretakers also stated jaundice, vomiting, diarrhea, convulsions and skin infection as signs and symptoms that would prompt them to bring the infant to a health facility. Regarding the duration of waiting time at PPHI health facilities, only one (4%) caretaker perceived waiting time to be long, 13 (50%) perceived it as acceptable and 12 (46%) perceived it as short. When asked about their perceptions of quality of services provided, 23 (88%) caretakers perceived the services as good, one (4%) felt a need for improvement, whereas two (8%) declined to offer any opinion on this subject.

## Discussion

Our survey evaluated the quality of management of sick young infants in BHU plus facilities based in peri-urban and rural areas in a programmatic setting, where PSBI guidelines were scaled up. We found that health facilities were well equipped with antibiotics and other commodities to manage sick young infants with PSBI at the time of the survey however, nearly a fifth of the facilities (17%) had a stockout at least once within a month prior to the survey. Ambulance services were available in 60% of the facilities [15]. We found at least one trained HCP in PSBI management who managed sick infants at each surveyed health facility. However, less than half the trained HCPs received their training within a year prior to the survey. It was also noted that except for two HCPs, all frontline providers were medical officers/physicians. Furthermore, two thirds of the health facilities were visited by a clinical supervisor in the previous six months.

Our data reported a high level of satisfaction of caregivers (88%) with the health care services provided to their sick infants, which was either comparable or higher than that reported from Nepal (82%)[16], South Korea (75%) [17], Ethiopia (63%) [18] and Ghana (50%) [19]. Results were mixed for counselling provided to care-takers by the HCPs in our survey. Most caretakers were counselled by the HCPs to breastfeed the infant and to bring the infant back if condition worsened, however, only a quarter were counseled about danger signs in detail. Only half the caretakers were advised to keep the infant warm, which could be attributed to the atmospheric temperatures in interior Sindh in the summer season (time of the survey) which can rise as high as 50°C during the day.

In general, the sick newborn and young infant care is sub-optimal at the primary health care level in Pakistan and many of them are referred to higher level health facilities as reported by a nationwide study [20]. It reported that knowledge of physicians working at similar settings in Pakistan regarding maternal and neonatal health was 53%, whereas only 22% were competent in basic newborn care and 47% could counsel mothers adequately [20]. In comparison, the observed counselling and communication skills of HCPs in our survey were higher, although it still needed improvement in several areas. Nearly all HCPs in our survey counselled caretakers on exclusive breastfeeding for young infants, which was higher than that reported from Nepal (15%) [16] and seven African countries (10%) [21].

Our data shows that for young infant and newborn care, availability of drugs, supplies and equipment and overall counselling of caretakers was better when compared with other health facility assessments in Ghana [19], Pakistan [22] and Uganda [23]. Our data also revealed better overall service provision compared to an assessment of PHC facilities in ten LMICs evaluated services available for sick children according to IMCI guidelines [24]. Health facility surveys conducted in the past have focused on care during pregnancy and delivery, and essential newborn care [22, 24, 25], whereas we focused on PSBI management of young infants.

The lessons learnt from this survey has program implications for PPHI, which need to be addressed. A critical finding was sub-optimal clinical management of sick young infants, especially those categorized as clinical severe infection who are at a higher risk for adverse outcomes. Some essential elements such as assessments for danger signs should be carried out every sick young infant visiting a health facility [13]. All danger signs were assessed in a very small proportion of infants, which is concerning since HCPs could have easily missed out critically sick infant. Moreover, for the infant identified with CSI who accepted referral, pre-referral antibiotic was not given which can prove life-threatening if not treated in a timely manner [26]. The most worrying aspect was inadequate antibiotic regimen treatment of most CSI cases by HCPs despite the availability of antibiotics, IMCI chart booklets and other job aids. This could be due to a few reasons. First, more than 40% HCPs were trained more than a year ago without any refresher training afterwards. Second, prior to implementation of PSBI guidelines, sick young infants were mostly referred to a higher level facility for inpatient care from these primary care facilities due to national guidelines so most of families with sick infant sought care directly from higher level health facilities or private providers, thereby resulting in loss of skills by the HCPs by lack of practice. Third, we found that nearly four-fifths of HCPs referred to job aids in the presence of the surveyors, but still failed to assess infants for all danger signs and did not manage CSI cases accurately indicating that they were clearly not routinely utilizing the job aids effectively. Finally, PPHI supervisors, who visited the health facilities did not always focus on young infant care. However, it is important to note that our assessment is contingent on a very limited number of CSI cases that were identified during our survey. In order to address these concerns, strict supervision and clinical monitoring should be in placed to assess adherence to management provided by the HCPs. A similar scale-up of PSBI guidelines in Bangladesh saw a relatively better antibiotic treatment of young infants identified with PSBI, where 44.5% of infants with CSI and 82.6% of infants with pneumonia were correctly classified and treated by HCPs [27].

It is well documented that IMCI trained workers were more likely to correctly classify and manage illnesses and have an impact on child health outcomes [28–30]. We believe that the quality of HCPs performance can be improved by refresher trainings, better supportive supervision including clinical mentoring and group work between trained health workers to solve issues [31, 32]. In addition, the counselling of the caretakers by HCPs was inadequate in several aspects. IMCI guidelines recommend counselling by HCPs at the individual level, peer-counselling at home and social mobilization through community leaders [33]. Literature has suggested that for parents and community members to be more aware of danger signs in infants and children, counselling and awareness sessions could be conducted during waiting times at health facilities, and during antenatal and postnatal care visits [34]. Furthermore, LHWs can also play an integral role to improve knowledge in communities about danger signs and empower families to make early care seeking decisions since most of the health facilities surveyed were affiliated with LHWs [35–38]. LHWs have the potential to act as catalysts for improved newborn care and improve young infant survival through early identification and timely referral to the health facilities, as shown in previous South Asian studies [3, 39].

The strength of our data is that it is the first health facility evaluation undertaken in Pakistan to evaluate the quality of young infant care and PSBI case management when referral is not feasible. The results of this evaluation will facilitate implementation and scaling up PSBI guidelines in other parts of the country and improve the future implementation with lessons learnt here. We acknowledge a few limitations in our survey. First, our limited resources did not allow us to extend this evaluation more widely over a longer time period to capture more cases as relatively small numbers of sick young infants were identified during the survey. Second, HCPs were aware of being observed, and it might have resulted in an attempt to portray an ideal picture of their services and facility, leading to response bias. Third, we neither used service provision assessment tools nor did we conduct an evaluation of the overall health system (infrastructure, outreach services and coherence in services) in our survey [22, 24].

## Conclusion

Scaling up such an intervention in sick young infants is valuable for any setting where acceptance of referral advice to higher level health facilities is a major challenge to appropriate management of neonatal sepsis. It will allow sick infants with PSBI who present at primary health care facilities to be managed at the first point of care when referral is not feasible, thus increasing access to treatment and saving lives. Despite the availability of commodities and provider knowledge, the adherence to guidelines in clinical practice was sub-optimal, which is of real concern. It is imperative that health system support at all levels is essential for successful scale-up. Particularly focus should be paid on refresher trainings of trained care providers and their supportive supervision, mentoring and vigorous monitoring and regular facility audits. It is also essential to establish referral linkages with the higher health facilities; and most importantly trust of communities in the quality of services being offered at public health facilities.
